# Emotional stress-induced hot phases in a patient with a desmoplakin-associated arrhythmogenic cardiomyopathy: a case report

**DOI:** 10.1093/ehjcr/ytag588

**Published:** 2026-11-04

**Authors:** Annalisa Caputo, Lucia Ilaria Birtolo, Elena Sofia Canuti, Gianmarco Scoccia, Carmine Dario Vizza

**Affiliations:** Department of Medical and Cardiovascular Sciences, Policlinico Universitario Umberto I, Sapienza University of Rome, viale del Policlinico 155, 00161 Rome (RM), Italy; Department of Medical and Cardiovascular Sciences, Policlinico Universitario Umberto I, Sapienza University of Rome, viale del Policlinico 155, 00161 Rome (RM), Italy; Cardiology SS. Annunziata Hospital ASL CN1, Via Ospedali 9, 12038 Savigliano (CN), Italy; Department of Medical and Cardiovascular Sciences, Policlinico Universitario Umberto I, Sapienza University of Rome, viale del Policlinico 155, 00161 Rome (RM), Italy; Department of Medical and Cardiovascular Sciences, Policlinico Universitario Umberto I, Sapienza University of Rome, viale del Policlinico 155, 00161 Rome (RM), Italy

**Keywords:** Arrhythmogenic cardiomyopathy, DSP-associated cardiomyopathy, Adrenergic stress, Cardiac magnetic resonance, Hot phase myocarditis, Case report

## Abstract

**Background:**

Desmoplakin (DSP) mutations are a common cause of arrhythmogenic cardiomyopathy (ACM), frequently presenting with recurrent myocarditis-like ‘hot phases’ that contribute to myocardial injury and increased arrhythmic risk.

**Case Summary:**

A 40-year-old woman presented with acute chest pain and marked troponin elevation following emotional stress. Her history was notable for recurrent stress-related myocarditis episodes and a diagnosis of biventricular arrhythmogenic cardiomyopathy due to a pathogenic DSP mutation. Cardiac magnetic resonance demonstrated diffuse myocardial oedema and a characteristic circumferential subepicardial ring-like pattern of late gadolinium enhancement, consistent with an active hot phase. Given the high-risk pathogenic DSP mutation with multiple high-risk features, including documented non-sustained ventricular tachycardia, frequent ventricular ectopy, and mild left ventricular disfunction (resulting in a calculated 5-year arrhythmic risk of 12% according to the novel DSP risk score)—and the presence of frequent hot phases recurrences and high burden of late gadolinium enhancement—a primary prevention implantable cardioverter–defibrillator was implanted after clinical stabilization. Medical therapy was optimized with β-blocker up-titration and low-dose selective serotonin reuptake inhibitor therapy targeting recurrent stress-related triggers. At follow-up, the patient reported sustained clinical well-being with no recurrence of hot phases or arrhythmic events.

**Conclusion:**

This case exemplifies DSP-related arrhythmogenic cardiomyopathy marked by recurrent, stress-triggered ‘hot phases,’ highlighting the pivotal interplay between inflammation and arrhythmic risk, and underscoring the need for refined, disease-specific risk stratification and targeted management strategies.

Learning points

- DSP-related ACM is marked by recurrent myocarditis-like ‘hot phases’, reflecting inflammatory and adrenergic drivers of myocardial injury and arrhythmic risk.
- Adrenergic stress may trigger disease flares, supporting β-blockade and adjunctive strategies to prevent recurrence.
- Integrated phenotypic assessment (genotype, CMR, triggers) is key for accurate risk stratification and personalized management.

## Introduction

Desmoplakin (*DSP*)-associated arrhythmogenic cardiomyopathy (ACM) is a distinctive phenotype characterized by recurrent myocarditis-like episodes (‘hot phases’), extensive myocardial fibrosis, and an increased risk of ventricular arrhythmias. These inflammatory flares are increasingly recognized as important drivers of myocardial injury and disease progression, although the mechanisms triggering their occurrence remain poorly understood. We describe a patient with DSP cardiomyopathy who experienced recurrent hot phases consistently following intense emotional stress, highlighting a possible link between adrenergic activation, myocardial inflammation, and arrhythmic risk.

## Summary figure

**Figure:**
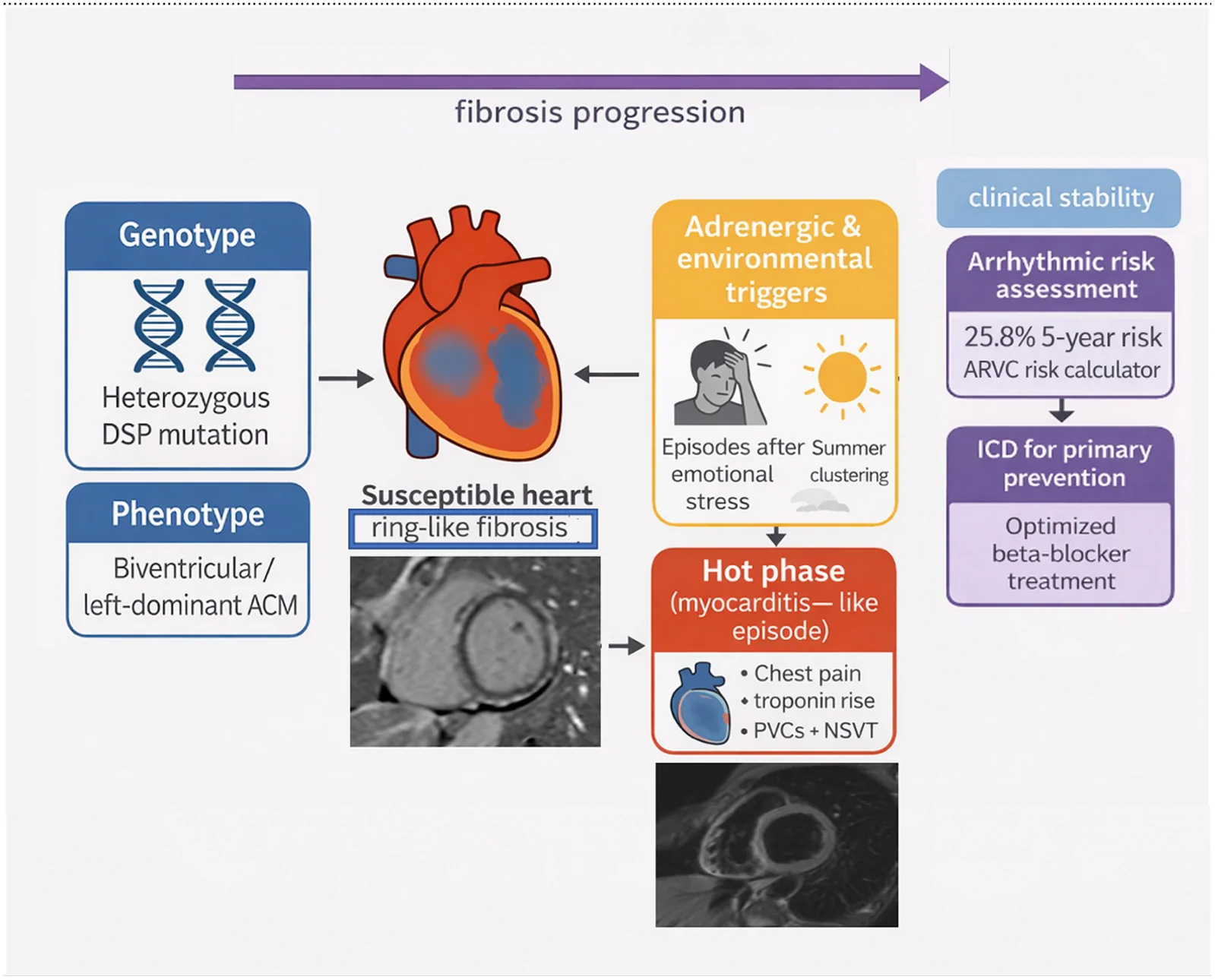
Pathophysiological framework and clinical course of DSP-associated arrhythmogenic cardiomyopathy. Schematic representation of the interplay between genetic substrate, environmental triggers, and disease progression in DSP-related arrhythmogenic cardiomyopathy (ACM). A heterozygous DSP mutation underlies a biventricular/left-dominant ACM phenotype characterized by a circumferential subepicardial ‘ring-like’ fibrosis on cardiac magnetic resonance. Superimposed adrenergic and environmental stressors—particularly emotional stress and seasonal factors—precipitate recurrent inflammatory ‘hot phases’ (myocarditis-like episodes) presenting with chest pain, troponin elevation, and increased arrhythmic burden. These episodes contribute to progressive fibrotic remodelling and cumulative myocardial damage over time. Comprehensive arrhythmic risk stratification (estimated 5-year arrhythmic risk approximately 12% according to the DSP risk score) guided primary prevention implantable cardioverter–defibrillator implantation and therapeutic optimization, resulting in subsequent clinical stability.

## Case presentation

A 40-year-old Caucasian woman presented to the emergency department with acute retrosternal chest pain, dyspnoea, and profound fatigue occurring 48 h after a highly stressful professional event. She denied fever, recent respiratory or gastrointestinal symptoms, vaccination, or recent travel. Her cardiovascular risk profile was unremarkable. The medical history was notable for recurrent episodes of myocarditis since 2007. These events consistently followed periods of intense emotional or physical stress (e.g. occupational examinations, family bereavement) and frequently clustered during summer months. In 2021, after a further episode, she was diagnosed with biventricular arrhythmogenic cardiomyopathy (ACM) based on cardiac magnetic resonance (CMR) findings and confirmatory endomyocardial biopsy demonstrating fibrous myocardial replacement. Genetic testing identified a heterozygous pathogenic mutation in the *desmoplakin* (DSP) gene (c.3337C > T, p.R1113X). Cascade screening revealed the same variant in her father (asymptomatic) and paternal uncle (single prior myocarditis episode).

At presentation, she was receiving bisoprolol 1.25 mg daily; previous attempts at higher doses had resulted in marked sinus bradycardia with a 4-s asymptomatic pause. On admission, she was haemodynamically stable. Electrocardiography demonstrated sinus rhythm (60 bpm), first-degree atrioventricular block (PR 220 ms), low voltages in limb leads and fragmented QRS complexes in inferior and lateral leads (*Figure 1*). High-sensitivity troponin T was markedly elevated (0.32 μg/L; x20 upper limit of normal), while inflammatory markers remained within normal ranges. Chest radiography showed mild cardiomegaly. Transthoracic echocardiography revealed normal left ventricular (LV) dimensions with lower-normal systolic function (LVEF 53%), regional hypokinesia of the basal inferior and inferolateral segments, preserved right ventricular (RV) function, and a small pericardial effusion.

**Figure 1:**
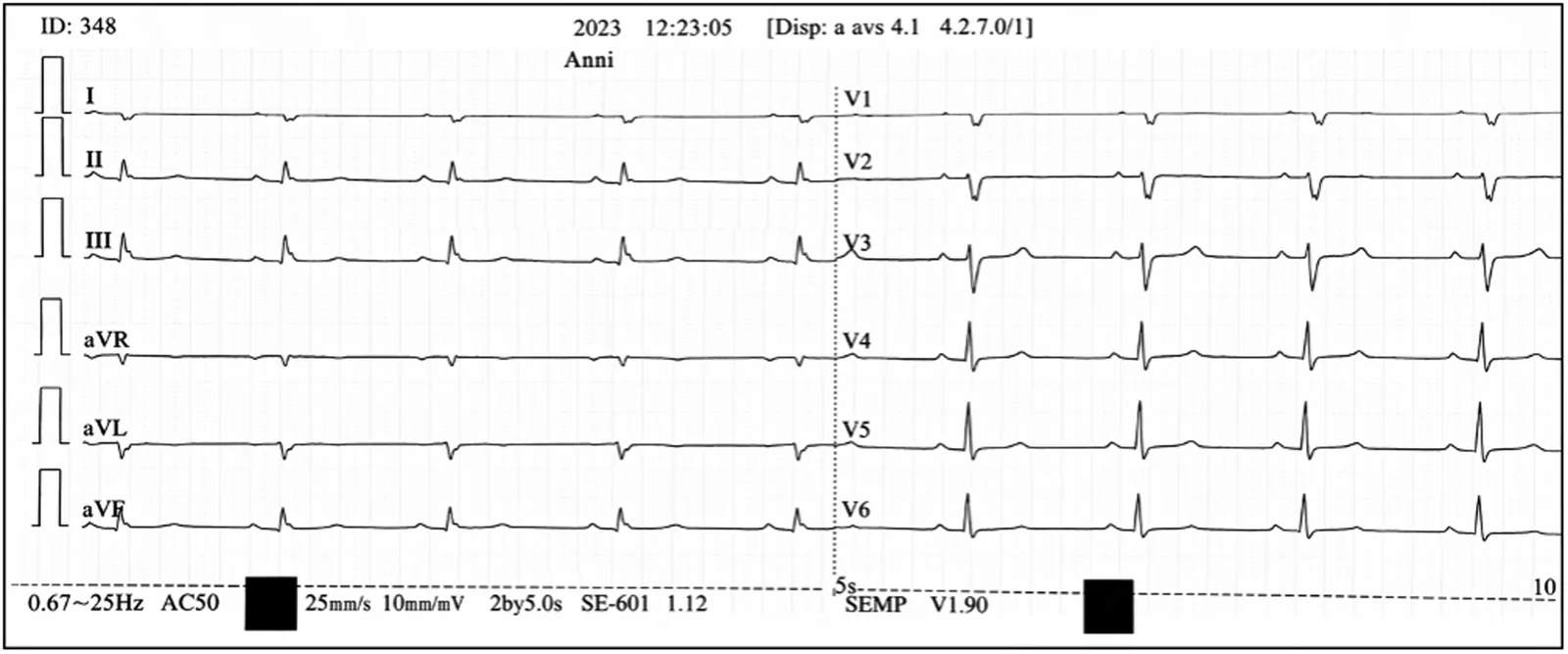
Electrocardiogram performed at ICU admission. Electrocardiography demonstrated sinus rhythm (60 bpm), first-degree atrioventricular block (PR 220 ms), low voltages in limb leads and fragmented QRS complexes in inferior and lateral leads.

Continuous telemetry recorded >2000 premature ventricular complexes and one episode of non-sustained ventricular tachycardia (eight beats). Contrast-enhanced CMR demonstrated preserved LV volumes with mildly reduced systolic function (LVEF 52%; RVEF 46%). Late gadolinium enhancement (LGE) showed a circumferential subepicardial ‘ring-like’ pattern with mesocardial septal involvement and extension to the inferior RV wall (*Figure 2A* and *B*). No fatty infiltration was observed. T2-weighted imaging revealed diffuse myocardial oedema involving the LV anterolateral, inferior, and inferolateral walls and the basal RV (*Figure 2C*).

**Figure 2:**
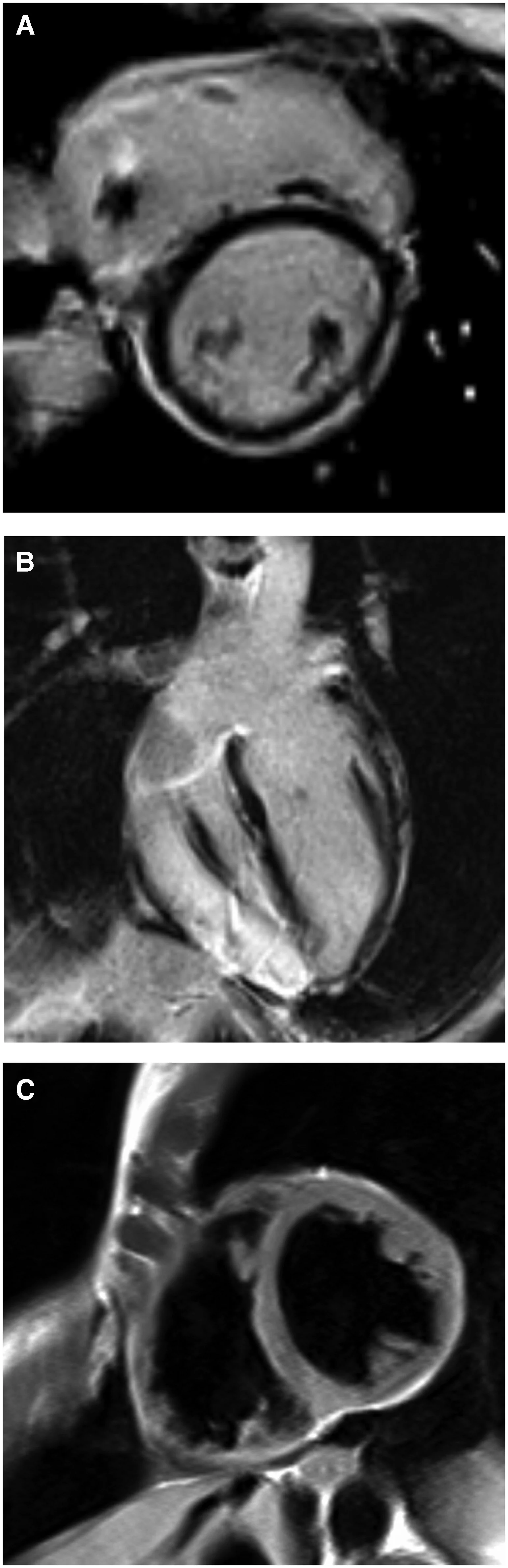
Contrast enhanced cardiac magnetic resonance performed during hospitalization for hot phase. (*A*, *B*) Late gadolinium enhancement (LGE) sequences in short-axis view, showing a ring-like pattern of LGE (*A*) and four-chamber view (*B*). (*C*) T2-weighted STIR images in short-axis view, demonstrating myocardial oedema.

A contrast-enhanced CMR performed 3 months earlier during a clinically quiescent phase had demonstrated persistent subepicardial ring-like fibrosis without oedema, underscoring the dynamic inflammatory component of the current episode.

Given the high-risk pathogenic DSP mutation with multiple high-risk features, including documented non-sustained ventricular tachycardia, frequent ventricular ectopy, and mild LV disfunction (resulting in a calculated 5-year arrhythmic risk of 12% according to the novel DSP risk score)—and the presence of frequent hot phases recurrences and high burden of LGE, a primary prevention transvenous implantable cardioverter–defibrillator (ICD) was implanted. This strategy permitted cautious up-titration of β-blocker therapy. In view of the consistent stress-related triggers of inflammatory ‘hot phases’, propranolol was introduced and low-dose selective serotonin reuptake inhibitor (SSRI) therapy initiated to mitigate stress-related adrenergic surges.

The patient was discharged in stable condition. At 6-month follow-up, she remained free from chest pain and clinically significant arrhythmias. Biomarkers normalized, and echocardiography demonstrated stable biventricular systolic function. Follow-up CMR showed resolution of oedema, mildly improved biventricular function (LVEF 56%; RVEF 52%), and persistent subepicardial fibrosis. ICD interrogation revealed no sustained ventricular arrhythmias. At 1- and 2-year follow-up, she reported sustained clinical stability without recurrence of inflammatory episodes or arrhythmic events.

## Discussion

This case illustrates DSP-associated ACM, a phenotype characterized by recurrent ‘hot phases’—myocarditis-like episodes that accelerate myocardial injury and markedly increase the risk of ventricular arrhythmias.^1^

Increasing evidence indicates that ACM arises from a complex interplay between genetic susceptibility, inflammatory pathways, and immune dysregulation. In this context, hot phases represent acute inflammatory flares superimposed on a vulnerable myocardial substrate. They are most frequently linked to DSP mutations but have also been described with PKP2, DSG2 and other cardiomyopathy-related genes. This ‘second-hit’ mechanism involves desmosomal dysfunction, impaired cell adhesion and dysregulated Wnt/β-catenin and Hippo/YAP signalling, which render cardiomyocytes more susceptible to exogenous stressors such as adrenergic stimulation, viral infection or autoimmunity.^2–4^ Histopathology and experimental models demonstrate inflammatory infiltrates, cytokine release, and complement activation preceding overt ventricular dysfunction,^5^ supporting the view that inflammation directly contributes to myocyte loss, fibrotic remodelling, and arrhythmogenesis.^6^

In our patient, ‘hot phases’ occurred in the absence of infectious prodromes, while environmental and adrenergic triggers appeared central, with each episode arising after significant emotional stress and typically during the summer months. Excess β-adrenergic stimulation—such as that associated with high-intensity exercise—has been shown to dysregulate intracellular calcium handling, activate inflammatory cascades, and promote oxidative stress.^7^ Preclinical studies in PKP2-deficient murine models demonstrated that exercise-induced adrenergic drive can trigger intracellular β-adrenergic receptor activation and phospholamban hyperphosphorylation, thereby amplifying arrhythmogenic burden.^8^ Consistently, clinical data from Saberniak *et al*. and others show that athletes with ARVC/D exhibit more pronounced biventricular dysfunction compared with non-athletes, and that high-intensity exercise is linked to earlier disease manifestation, increased risk of malignant arrhythmias, and sudden cardiac death.^6,9^ Taken together, these findings support the hypothesis that psychological stress–related adrenergic hyperactivation may, through similar mechanisms, also contribute to myocardial injury in ARVC/D; however, direct evidence for this association remains limited. In this context, β-blockers—particularly membrane-permeable agents such as propranolol—may mitigate adrenergic injury and inflammation.^10^ In view of the patient’s history of recurrent ‘hot phases’ apparently triggered by stress-related adrenergic hyperactivation, therapy was optimized during hospitalization with the introduction of propranolol and a mild anxiolytic strategy using a low dose SSRI. This combined approach was associated with a reduction in anxiety symptoms and improved overall clinical stability at follow-up, with no further ‘hot phases’ reported.

DSP cardiomyopathy is strongly associated with ventricular arrhythmias and sudden cardiac death, even in the absence of overt ventricular dysfunction. In large series, approximately one quarter of DSP cardiomyopathy patients experience sustained ventricular arrhythmias or sudden cardiac death during follow-up.^11^ However, the traditionally employed ARVC risk calculator has proven inadequate in DPM-ACM,^12^ making risk stratification challenging. According to latest evidence, the presence of prior non-sustained ventricular arryhtmia, LVEF ≤ 50%, and previous myocardial injury events are amongst the most prominent risk features for adverse events in DSP cardiomyopathy,^13^ while history of recurrent myocarditis-like episodes appears to further increases the overall risk.^14^ Recently, a DSP specific risk calculator has been proposed, based of five clinical factors: Female sex, presence of non-sustained ventricular tachycardia, number of 24-h premature ventricular contraction burden, right ventricular systolic dysfunction, and left ventricular ejection fraction^15^ In our patient, given the moderate-high 5-year arrhythmic risk (approximately 12% according to the DSP risk score), in addition to the presence of a high-risk genetic mutation, frequency of hot-phase episodes and presence of extensive LGE, a transvenous ICD was implanted for primary prevention of sudden cardiac death and to allow safe β-blocker up-titration. The coexistence of sinus bradycardia, first-degree AV block, and marked sensitivity to low β-blocker doses further favoured a transvenous over a subcutaneous device, ensuring pacing activity if needed.

This case underscores the complexity of DSP-associated arrhythmogenic cardiomyopathy, a condition in which the underlying pathophysiological mechanisms remain only partially elucidated.

Increasing evidence suggests that genetic predisposition, inflammatory processes, and especially adrenergic stress converge to drive myocardial injury, yet the precise triggers and molecular pathways are still being defined. In our patient, recurrent ‘hot phases’ were closely linked to episodes of marked emotional and adrenergic activation, with a seasonal clustering in the summer months that may further amplify adrenergic drive. Recognizing the central role of adrenergic hyperactivation and promptly optimizing medical therapy—including β-blocker titration and low-dose SSRI administration—proved essential in reducing the recurrence of hot phases. Because these inflammatory flares are closely linked to cumulative myocardial damage and a higher arrhythmic burden, their prevention is a critical therapeutic goal. This case therefore highlights the importance of a holistic approach combining adrenergic modulation, risk stratification, lifestyle modification, and targeted pharmacotherapy to mitigate disease progression and improve outcomes in DSP-related ACM.

## Lead author biography


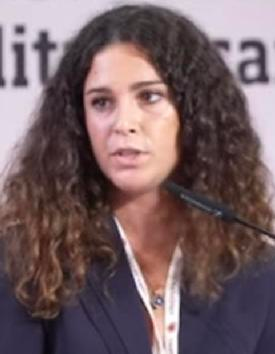
Dr Annalisa Caputo is a cardiologist and PhD candidate in Cardiothoracic and Vascular Pathophysiology and Imaging at Sapienza University of Rome. Her research focuses on advanced cardiac imaging, with particular interest in its application to pulmonary hypertension and acute and chronic heart failure.

## Data Availability

All data used in this study are publicly available and can be accessed through the references cited in the bibliography, including the corresponding DOI links.
